# Substance Use Among Youth During the COVID-19 Pandemic: a Systematic Review

**DOI:** 10.1007/s11920-022-01338-z

**Published:** 2022-04-27

**Authors:** Hannah M. Layman, Ingibjorg Eva Thorisdottir, Thorhildur Halldorsdottir, Inga Dora Sigfusdottir, John P. Allegrante, Alfgeir Logi Kristjansson

**Affiliations:** 1 Department of Social and Behavioral Sciences, School of Public Health, West Virginia University, Morgantown, WV USA; 2 Department of Psychology, Reykjavik University, Reykjavik, Iceland; 3 Icelandic Centre for Social Research and Analysis, Reykjavik University, Reykjavik, Iceland; 4 Department of Health and Behavior Studies, Teachers College, Columbia University, New York, NY USA; 5 Department of Sociomedical Sciences, Mailman School of Public Health, Columbia University, New York, NY USA

**Keywords:** Adolescents, COVID-19, Drug abuse, Pandemic, Substance use, Systematic review

## Abstract

**Purpose of Review:**

To review the literature on the trends in substance use among youth during the coronavirus SARS-CoV-2 (COVID-19) pandemic.

**Recent Findings:**

The pandemic has given rise to concerns about the mental health and social well-being of youth, including its potential to increase or exacerbate substance use behaviors. This systematic review identified and included 49 studies of use across alcohol, cannabis, tobacco, e-cigarettes/vaping, and other drugs, and unspecified substances. The majority of studies across all categories of youth substance use reported reductions in prevalence, except in the case of other drugs and unspecified drug and substance use, which included three studies that reported an increase in use and three studies that reported decrease in use.

**Summary:**

Overall, the results of this review suggest that the prevalence of youth substance use has largely declined during the pandemic. Youth substance use in the post-pandemic years will require monitoring and continued surveillance.

**Supplementary Information:**

The online version contains supplementary material available at 10.1007/s11920-022-01338-z.

## Introduction

The adolescent years represent an important developmental stage during which the foundation for future patterns in substance use is often established [1]. Both the quantity and frequency of use during this period are strongly associated with risks for heavy use and misuse of substances in adulthood [2, 3]. As an example of the staggering economic and societal costs, substance use in the USA alone has been estimated at over $400 billion annually by the US Surgeon General [4]. In addition to the direct economic impact, the societal harm caused by substance use in the USA has been estimated at over $800 billion annually due to premature death or quality-of-life adjustments [5]. Youth alcohol, tobacco, and other drug use impairs psychological and neurocognitive development and increases risk for academic failure, chronic disease, and mental illness [6, 7]. Thus, the prevention of youth substance use remains an important priority for public health globally.

Various domains of established risk and protective factors play an important role in preventing the development of youth substance use. These include access to care and support provided by parents, family, and friends; structure, supervision, and support from school faculty and staff; and access to and participation in pro-social leisure time activities [8, 9]. Studies that take an ecologic view of substance use have further assessed the impact of environmental factors known as “context effects,” which independently contribute to the odds of alcohol, tobacco, and other drug use among youth. Generally, such studies have found that youth who live under challenging home situations or in resource-limited areas, or both, are more likely than other youth to be negatively affected by sudden environmental changes and thus may turn to substance use as a coping mechanism [10–12].

### The COVID-19 Pandemic

The novel coronavirus SARS-CoV-2 (COVID-19) was officially declared a pandemic by the World Health Organization (WHO) on March 11, 2020 [13]. Over 400 million confirmed cases and close to 6 million deaths worldwide have been attributed to the virus [14]. Thus, virtually no human on earth has been unaffected by the virus. During this time, entire countries, regions, states, cities, and towns have enacted various laws, rules, and guidelines in their efforts to curb the spread of the virus and its impact on human health. Some of the more drastic mitigation measures have included closing of borders, lockdowns and curfews, or both, in cities and towns; severe limits on social gatherings and assembly (e.g., religious services); restricted access to worksites and entertainment venues and services (e.g., restaurants, theaters, and sports events); and mandates for physical (or social) distancing and wearing face masks. In most places, these efforts have included closing of schools and restriction of services for youth, such as sport clubs and extracurricular programs, and the prohibition of social gatherings [15, 16]. Such extreme measures at the societal level are unprecedented in modern times and have not been seen since the influenza pandemic of 1918 [17].

In addition to the social restrictions, the mitigation efforts to curb the spread of the virus have resulted in unintended consequences that have been harmful in the lives of youth [18]. These include disruption of parental (or caregiver) income and associated financial consequences and stunted academic progress due to school closings, remote instruction, and recurring changes in instructional formats. The pandemic has also increased feelings of loneliness among young people because of long-term social isolation and limited opportunities to interact with peers [12]. During this period, inconsistent and poorly planned institutional responses have been reported [19], including a decline in access to harm-reduction services and treatment of substance use [20]. In a recent review, Pfefferbaum highlighted the negative psychological effects of the pandemic on children and youth, including the significant increase in the prevalence of clinical depression, suicidal ideation, and anxiety, all of which have the potential to contribute to an increase in substance use behaviors [21].

### The Current Study

Given the human and societal costs associated with youth substance use, we sought to critically assess the impact that the COVID-19 pandemic has had on youth substance use. Some recent studies have shown an increase of substance use among youth, particularly vulnerable youth, such as those living in resource-poor areas or under challenging family circumstances [22], while others have found a reduction in substance use despite an overall worsening of mental health status [23••]. However, despite the significance of the pandemic, a wholistic review of research on youth substance use during the era of the COVID-19 pandemic has not been conducted to date. Consequently, the objective of this systematic review was to provide an overview of the most recent research into youth substance use during the period of the COVID-19 pandemic.

## Methods

This systematic review sought to examine the prevalence of substance use among adolescents during the COVID-19 pandemic. Following the identification and selection of peer-reviewed papers, we examined each relevant paper by country, sample characteristics (type, age, sample size, period of study enrollment), study design, substance use behavior or outcome (type, measurement), and covariates included in the analyses. The Preferred Reporting Items for Systematic Reviews and Meta-analysis (PRISMA) was used to guide the design, execution, and reporting of findings for this systematic review. The research question, inclusion criteria, and search terms were defined using the PICO approach (Population, Intervention [or Exposure], Comparator, and Outcome). We identified and used previously published research articles and reviews on substance use during the COVID-19 pandemic to guide the creation of the search terms. The protocol for this systematic review was registered at PROSPERO (CRD42022311679).

### Inclusion and Exclusion Criteria

Studies were selected based on the following criteria: (1) examined the substance use among youth during the COVID-19 pandemic; (2) study participants were 24 years old or younger; and (3) the study was published in the English language. Cross-sectional and longitudinal studies were included. When two manuscripts presented findings from non-independent datasets, the manuscript with the larger number of study participants was included. Articles were excluded if either COVID-19 (or a related term: COVID pandemic, Coronavirus, etc.) or substance use (or related terms: substance abuse, addiction, alcohol, nicotine, smoking, vaping, tobacco, licit drug/s, illicit drug/s, drug/s, etc.) was not identified in the paper’s title or abstract.

### Identification of Studies

All databases within Web of Science were used in conducting the search. The search was limited to studies published on, or subsequent to, the date the COVID-19 pandemic began (December 1, 2019) to studies published up to February 15, 2022. Thus, the last search for this review was conducted on February 15, 2022. Titles, abstracts, and articles were reviewed to identify potentially relevant manuscripts. The search terms included combinations of COVID, adolescent*, child*, youth, substance use, substance abuse, drug, substance drug, smoking, tobacco use (Table 1). Reference lists of included research studies and published reviews of substance use among youth during the COVID-19 pandemic were also searched.

**Table 1:** Search terms and linkage (Web of Science)

Concept	Search term
Exposure	COVID
	AND
Participants	youth OR adolescent OR adolescence OR children
	AND
Outcomes	substance use OR substance abuse OR drug OR smoking OR tobacco use

### Data Extraction

The initial search based on the inclusion and exclusion criteria was performed by one investigator (HL) and then repeated by a second investigator (IET) to ensure that all relevant articles were included. The investigators conducting the search were located across two different countries (the USA and Iceland) with access to different research databases. As such, the second investigator’s search yielded an additional 17 studies that were not included in the first search. These discrepancies in the search findings from the two investigators who performed the search were discussed and a consensus was reached by the two investigators. Key elements of relevance for this review were extracted from each paper, summarized, and entered into an Excel spreadsheet, which was used to inform the broader discussion of the current state of the literature among the collaborating authors.

## Results

**Fig. 1:**
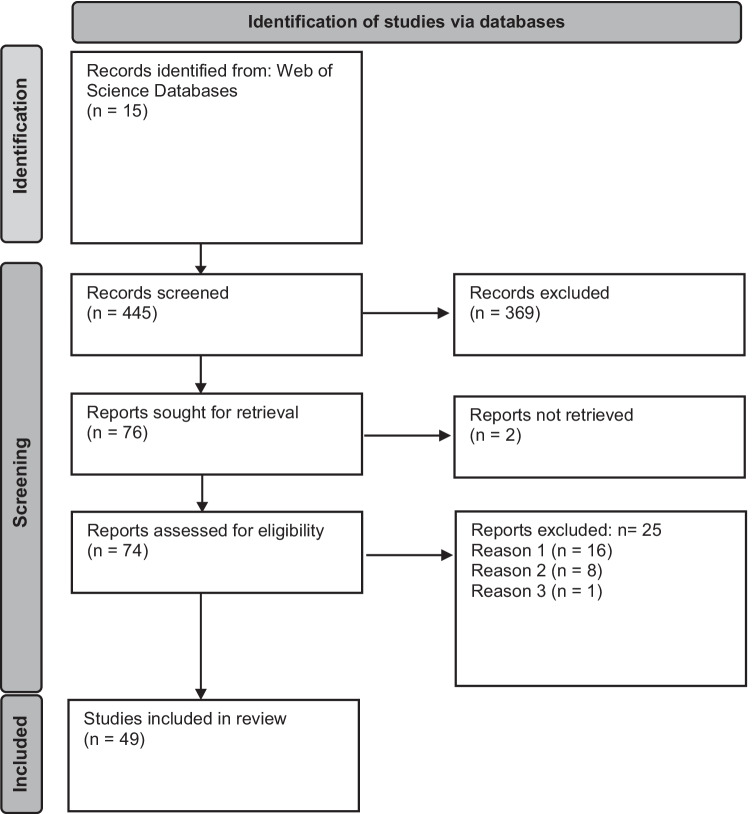
PRISMA flow diagram of the bibliographic search. The 15 Web of Science databases included: Arts & Humanities Citation Index, Book Citation Index, Emerging Sources Citation Index, BIOSIS Citation Index, BIOSIS Previews, Conference Proceedings Citation Index, Data Citation Index, Derwent Innovations Index, KCI-Korean Journal Database, MEDLINE®, Russian Science Citation Index, Science Citation Index Expanded, Social Sciences Citation Index, SciELO Citation Index, Zoological Record, Zoological Record (1864-present). Reasons for excluding reports included the following: reason 1, accidentally included/wrong topic; reason 2, not a research article; and reason 3, date of publications prior to the COVID-19 outbreak

The initial search yielded 423 articles of potential interest. Of those, 49 articles met full eligibility criteria (see Fig. 1 for PRISMA flow chart). Five articles were published in 2020, 38 in 2021, and 6 in 2022. Forty-six articles from 23 countries reported on studies conducted with single-country samples and three articles reported on studies from multiple countries. Most of the studies were conducted in North America (*n* = 22) or Europe (*n* = 19). Twenty-nine articles reported studies that were based on cross-sectional designs and 20 on longitudinal designs. Forty-four articles reported on participant samples of between 10 and 25 years of age, and five articles also included older individuals. Regarding outcomes, 14 articles reported studies with a single substance use outcome, 29 articles included multiple substance use outcomes, five articles reported on general substance use without specifying type of substance, and one article focused solely on substance abuse disorder. Below, we have organized the summaries of our findings from the review of the 49 articles by *substance use outcome* (Table 2). Articles reporting on multiple substance use outcomes are included in multiple summaries based on the respective outcome.

**Table 2:** Description of studies included in the review and summary of findings

**Author(s)**	**Age**	**Sample size**	**Sample type**	Sample recruitment time/data collection*	**Cross-sectional (C) or longitudinal (L)**	**Type(s) of substance use**	**Substance use measurement**	**Main findings of relevance**
Albrecht et al. [38]	15–17	8,972	Voluntary response	May 2017–July 2017 & May 2020–June 2020	C	Alcohol and smoking (not specified)	Weekly alcohol consumption & smoker or non-smoker (only for participants 16 +)	Less alcohol consumption was reported during school closures
Ayran et al. [59]	19–24	503	Non-probabilistic: Purposive	May 2020 & June 2020	C	Nicotine	Fagerström Test for Nicotine Dependence	Higher levels of anxiety led to higher reporting of nicotine dependence in university students
Benschop et al. [39]	16–24	6,070	Convenience	May 2020 & October 2020	C	Tobacco, alcohol, cannabis, and other drugs (ecstasy (XTC/MDMA), amphetamines, cocaine, nitrous oxide, ketamine, LSD, psychedelic mushrooms/truffles, GHB, 2C-B, 3-MMC/4-MMC and/or any other drug)	Alcohol, tobacco and cannabis use in the last week, other drug use in the last month	There was an overall decrease or cessation in current use of substances, especially in drugs like ecstasy and nitrous oxide
Berki and Piko [40]	14–19	705	Voluntary response	December 2020	C	Tobacco, alcohol use, and drug abuse	International Health Behavior in School-aged Children (HBSC) survey	Although COVID caused mandatory isolation, many young people still engaged in substance use
Bourion-Bédès et al. [41]	17–25	3,764	Voluntary response	May 2020–May 2020	C	Alcohol, tobacco	Substance use reported during the pandemic (none, no change, increased consumption, reduced consumption)	Increased alcohol and tobacco consumption were identified as risk factors for high perceived stress
Branquinho et al. [69]	16–24	617	Convenience	April 2020–May 2020	C	Substance use (general)	No information provided other than the survey was designed for the study	An increase in substance use was reported
Branquinho et al. [70]	16–24	592	Convenience	February 2021–March 2021	C	Substance use (general)	No information provided other than the survey was designed for the study	Compared to girls, boys reported more negative consequences due to substance use
Chaffee et al. [24]	14–16	1,006	Non-probabilistic: Purposive	March–May 2019, August–February 2019, & September 2020	L	Alcohol, cigarettes, cigars, e-cigarettes, hookah, cannabis, and conventional smokeless tobacco	Ever used (yes/no) and number of days used (0–30) in the past	There was no significant change in the prevalence of e-cigarette, cannabis, or alcohol use
Chaiton et al. [25]	15–29	1,404	Voluntary response	November 2020–March 2021	C	Alcohol, cigarettes, cannabis, e-cigarettes, illicit drugs	Frequency of use in the past year	Participants in the study faced barriers to accessing mental health and addiction services as well as expressed need for additional supports
Chaiton et al. [26]	16–25	6,721	Voluntary response	August 2020–March 2021	C	Cigarettes, e-cigarettes, cannabis, alcohol	Rating of how the pandemic influenced the use of cigarettes, e-cigarettes, cannabis, and alcohol and their current use of each	An increase in the use of one or more substances during the pandemic was observed
Cho et al. [27]	14–21	2,120	Cluster sample	Fall 2013 (wave 1), Fall 2016 (wave 2), & May–August 2020 (wave 3)	L	Alcohol, combustible cigarettes, e-cigarettes, cigars/cigarillos, hookah, blunts, combustible cannabis, e-marijuana, marijuana edibles, dabbing, prescription stimulants, prescription pain-killers	Youth risk behavior surveillance survey (past 30-day substance use)	An escalation in substance use in young adults with prior emotional disturbances during the pandemic was observed
Clare et al. [51]	19–23	443	Voluntary response	September 2017–July 2018, September 2018–May 2019, August 2019–January 2020, & May2020–June 2020	L	Alcohol	Australian Parental Supply of Alcohol Longitudinal Study (APSALS)	A small reduction in alcohol use was observed during pandemic-related restrictions compared to before the restrictions were noted. There was also a large decline in alcohol-related harms during this same time
Clendennen et al. [54]	16–24	709	Voluntary response	Spring 2020	C	Marijuana, e-cigarettes, and cigarettes	National Survey on Drug Use and Health, the 10-item Hooked on Nicotine Checklist (HONC)	An increase or the same levels of current marijuana, e-cigarette, and cigarette use were reported during the pandemic
Dumas et al. [28]	14–18	1,054	Voluntary response	April 2020	L	Alcohol, vaping, cannabis, and binge drinking	2018 National Survey on Drug Use and Health	The percentage of young people binge drinking, vaping, and using cannabis (girls only) significantly decreased when comparing the pre-covid and post-covid social distancing orders; however, there was no significant change in the percentage of alcohol use
Dvorsky et al. [29]	15–17	238	Non-probabilistic: Purposive	May–June 2020, July–August 2020, & October–November 2020	L	Alcohol, vaping, and cigarettes	Substance use was rated on a five-point scale (1 = not at all; 5 = regularly), with higher scores indicating higher frequency of use of each type of substance	Adolescents with ADHD were at greater risk for experiencing increases in mental health symptoms and substance use throughout the pandemic, relative to adolescents without ADHD
Fruehwirth et al. [52]	18–20	439	Voluntary response	October 2019–February 2020 & June 2020–July 2020	L	Alcohol	Youth Risk Behavior Surveillance System	Alcohol use and binge drinking decreased
Gaiha et al. [62]	13–24	4,351	Voluntary response	May 2020	C	E-cigarette	National cross-sectional survey to assess e-cigarette use	Data showed that participants who have smoked cigarettes and do not believe that e-cigarette use increases their risk of contracting the COVID-19 virus were more likely to report using e-cigarettes in the past month
Gaiha et al. [63]	13–24	2,167	Voluntary response	May 2020	C	E-cigarette	Change in e-cigarette use, access to e-cigarettes before and after the COVID-19 pandemic began, reasons for change, number of times e-cigarettes were used, and nicotine dependence	Over half of the participants reported reducing e-cigarette use or quitting
Gesualdo et al. [49]	18–24	212	Voluntary response	November 2020–December 2020	C	Alcohol	Alcohol Use Disorders Identification Test	College students who moved away from home consumed more alcohol than college students who did not leave their home
Gilic et al. [30]	15–18	661	n/a	January 2020 & April 2020	L	Cigarette smoking, alcohol consumption, and drug consumption	Alcohol Use Disorders Identification Test	No change in smoking, alcohol consumption or drug use was observed during the pandemic
Hawke et al. [66]	14–28	622	Convenience	April 2020	C	Substance use in general	GAIN-Short Screener (GAIN-SS)	Substance use declined during the pandemic
Hawke et al. [31]	14–28	619	Convenience	April 2020, June 2020, August 2020, & October 2020	L	Substance use in general	GAIN-Short Screener (GAIN-SS)	Substance use remained stable during the pandemic
Hermosillo-de-la-Torre et al. [42]	14–21	8,033	Voluntary response	November 2020–December 2020	L	Alcohol, cannabis, tobacco, cocaine, inhalants, and methamphetamine	Problem-Oriented Screening Instrument for Teenagers (POSIT)	Alcohol and tobacco use indicated higher odds of suicidal behavior
Ho et al. [60]	15–25	201	Snowball	April 2020–May 2020	C	Smoking	Standardized and structured questionnaire covering smoking behavior, readiness to quit, and risk perceptions of smoking during the pandemic	The bulk of participants reduced their daily cigarette use and almost half of participants were motivated to quit
Hopkins and Al-Hamdani [64]	16–24	540	Non-probabilistic: purposive	April 2020–May 2020	C	E-cigarette	The 2020 Youth and Young Adult Survey	A decrease in the frequency of vaping and in puff per single vaping period was reported
Kerekes et al. [37]	15–19	5,114	Non-probabilistic: purposive	September 2020–February 2021	C	Cigarettes, alcohol	Changes concerning substance use	A significant decrease in alcohol use and intoxication during the pandemic compared to before was noted. There was no significant change in smoking
Kreslake et al. [65]	15–24	5,752	Convenience	January 2020–June 2020	C	E-cigarette	Self-reported change in the amount of e-cigarette use reported by current vape users during the COVID-19 pandemic	Due to stay-at-home orders, both difficulties in accessing e-cigarettes and a decrease in their use were noted during the pandemic
Kuitunen [67]	10–17	10,000	Convenience	January 2020–December 2020	L	Substance use (not specified)	Emergency Dept. nurse assessment (poisoning not included), breathalyzer (alcohol)	Overall incidence rates of intoxication among youth were higher during the pandemic than in the three previous years
Lansford et al. [43]	15–20	1,330	Non-probabilistic: purposive	2015–2016 & March 2020–January 2021	L	Cigarettes, alcohol, and illicit drugs	A 5-point scale rating from ‘decreased a lot’ to ‘increased a lot’ during the pandemic	Adolescents that reported poorer well-being before the pandemic were more likely to report an increase in substance use during the pandemic
Lazaro-Perez et al. [44]	18 +	310	Non-probabilistic: purposive	June 2020	C	Tobacco, alcohol, cannabis, cocaine, amphetamine-type stimulants, inhalants, sedatives or sleeping pills, hallucinogens, opiates, and other drugs	ASSIST questionnaire	A third of the university student participants had a high risk of tobacco and alcohol consumption
Leatherdale et al. [55•]	13–17	1,937	Non-probabilistic: purposive	2018, 2019, & May–July 2020	L	Cannabis	Past 12-month marijuana or cannabis use	No significant effect on young people’s cannabis use during the earlier stages of the pandemic was observed
Li et al. [45]	18–23	1,010	Voluntary response	December 2020–January 2021	C	Alcohol, cigarettes, other drugs	Frequency of substance abuse (0 to > 7 times a week)	Both male and female participants with anxiety problems were more likely to use drugs and alcohol
Martinez-Fernandez et al. [56]	14–16	21	Non-probabilistic: purposive	May 2020	C	Cannabis	The Spanish Survey on Drug Use in Secondary Education (ESTUDES) was used to measure cannabis use	During stay-at-home orders and other restrictions during the pandemic, young people commonly relied on their immediate network of friends to gain access to cannabis substances
Maurino et al. [46]	12–20	1,535	Voluntary response	August 2020–September 2020	C	Alcohol, tobacco, marihuana, other drugs	A semi-structured questionnaire with close-ended and open questions designed for the study	A decrease in substance use in all categories was reported
Merianos et al. [58]	18–24	756	Voluntary response	October 2020–December 2020	C	Exclusive, dual, and polytobacco e-cigarette	Self-report of substance use in the past 30 days	Participants who used two or more types of tobacco products were the most likely to report experiencing COVID-19 symptoms and they were at the highest risk to be diagnosed with COVID-19
Miech et al. [32•]	17–20	582	Simple random sampling	February–March 2020 & July–August 2020	L	Cannabis, alcohol, vaping	Monitoring the Future (MTF)	Despite no significant change of marijuana, alcohol, and vape use, perceived availability of these substances declined dramatically during the pandemic
Naguib et al. [33]	18–24	2,380	Cluster Sample	July 2020–October 2020	C	Cigarettes, alcohol, Tramadol, Heroin, Hashish, Bhang, Strox, Voodoo	Addiction Severity Index	During the pandemic, illicit substance users increased use by almost 80%
Pelham et al. [34••]	10–14	7,842	Simple random sampling	May 2021, June 2021, August 2021	L	Alcohol, cigarettes, e-cigarette, cigar/hookah/pipe, smokeless tobacco/chew/snus; cannabis (flower/concentrate/edible); prescription drugs not prescribed; used inhalants; any other drugs	Adolescent Brain Cognitive Development (ABCD) Study (past month use of each listed substance)	During the pandemic, a decrease in alcohol use was reported but an increase was reported in the use of nicotine and unprescribed prescription drugs
Pigeaud et al. [50]	< 18 (no range given; mean = 16)	482	Non-probabilistic: purposive	January 2019–December 2020	L	Alcohol	Acute Alcohol Intoxication diagnosis	A decrease in acute alcohol intoxication was reported
Roges et al. (2021) [35]	14–8	303	Convenience	October 2019–February 2020 & June–July 2020	L	Alcohol, cannabis, tobacco	DESK-COVID-Cohort survey, AUDIT-C test, CAST validated test (Cannabis Abuse Screening Test)	A general reduction in substance use during the pandemic was reported with the exception of vocational and educational training students who were at a higher risk of substance use than other students
Romm et al. [36]	M = 24.76 (no range given)	1,084	Convenience	September–December 2019 & March–May 2020	L	Cigarette, e-cigarette, marijuana, and alcohol	Past 30-day substance use frequency	Participants who reported increases in e-cigarette use and alcohol use were more likely to report adverse childhood experiences and depressive symptoms
Sen et al. [22]	10–20	2,932	Voluntary response	April–June 2020	C	Cigarettes, alcohol, illicit drugs	Alcohol Use Disorders Identification Test (AUDIT), Cigarette Dependence Scale 12 (CDS-12)	Over half of those who reported drinking alcohol reported increased drinking and over a third reported harmful or dependence-like drinking behavior. Adolescents who smoked reported decreased cigarette use. Over a third of adolescents who reported using drugs reported an increase in use
Singh et al. [68]	13–60	1,027	Voluntary response	December 1–31 2020	C	Alcohol, tobacco, and self-medication	Brief COPE	Significantly higher substance use was reported among young males compared to girls during the pandemic
Skumlien et al. [57]	16–30	798	Voluntary response	June–August 2020	C	Alcohol, cannabis, illicit drugs	Severity of Dependence Scale (Cannabis dependence)	An increase in alcohol use was reported during the lockdown among adolescents with a history of alcohol use. A decrease in the use of illicit drug use was noted among both adolescents with and without a history of such use. An increase in cannabis use was noted. No differences were noted in cigarette use
Thorisdottir et al. [23••]	13–18	59,701	Cluster sample	October or February in 2016 and 2018 & October, 2020	L	Cigarette, e-cigarette, and alcohol	Frequency of cigarette, e-cigarette, and alcohol use in the past 30 days	In the 15–18-year-old age group, substance use decreased with no differences by gender
Vera et al. [53]	18–25	305	Voluntary response	November 2019–February 2020 & March 2021	L	Alcohol	Daily Drinking Questionnaire (DDQ)	Alcohol use decreased during the pandemic, compared to before the pandemic
von Soest et al. [47]	13–18	227,258	Random sampling	Each Spring from 2014–2019, January–March 2020, January–March 2021	L	Tobacco, alcohol, cannabis	Nationwide Norwegian Survey	Alcohol and cannabis use decreased
Yu and Choe [48]	12–18	108,038	Stratified cluster	June to August in 2019 and August to November in 2020	C	Drinking, smoking	Korea Youth Risk Behavior Surveys (KYRBS)	A decrease in drinking and smoking was noted, with a larger decrease among adolescent boys than girls

^*^Month of administration reported when available. If not, the time of year or solely the year in some cases, as reported by the authors

### Alcohol Use

A total of 32 studies included measures on alcohol use; 27 of those also included measures on one or more other types of substance use [22, 23••, 24–31, 32•, 33, 34••, 35–48], with five focusing exclusively on alcohol use as the outcome [49–53]. Fourteen studies employed a cross-sectional design [22, 25, 26, 33, 37–41, 44–46, 48, 49] and 18 used longitudinal designs [23••, 24, 27–31, 32•, 34••, 35, 36, 42, 43, 47, 50–53]. Twenty-four studies used a non-random selection of participants, including convenience, purposive, or volunteer samples [22, 24–26, 28, 29, 31, 35–46, 49–53].

Five studies reported increase in alcohol use [22, 26, 30, 36, 45], 12 studies reported decrease in alcohol use [23••, 32•, 34••, 35, 38, 39, 47, 48, 50–53], and four studies reported no change [24, 28, 31, 43], as noted above, mainly because of cross-sectional design where alcohol was employed as a covariate or group divider. Eleven studies reported neither an increase nor a decrease in alcohol use [25, 27, 29, 33, 37, 40–42, 44, 46, 49]. Ten studies included a mention of gender [23••, 25, 28, 33, 41–43, 45, 46, 51], and five in relation to alcohol use [23••, 28, 33, 45, 51]. One concluded that boys [33] used more alcohol than girls during the pandemic, while two studies reported on greater increase in use among girls [28, 45]. No gender difference was reported in two of the studies [23••, 51].

### Cannabis Use

A total of 20 studies included measures on use of cannabis, including marijuana, hashish, and edibles. Seventeen of these also included measures into one or more other type of substance use [24–28, 31, 32•, 33, 34••, 35, 36, 39, 42, 44, 46, 47, 54], three of which focused exclusively on cannabis use as the outcome [55•, 56, 57]. Nine studies employed a cross-sectional design [25, 26, 33, 39, 44, 46, 54, 56, 57] and 11 used a longitudinal design [24, 27, 28, 31, 32•, 34••, 35, 36, 42, 47, 55•]. Fifteen studies used a non-random selection of participants, including convenience, purposive, or volunteer samples [24–26, 28, 31, 35, 36, 39, 42, 44, 46, 54, 55•, 56, 57].

Four studies reported an increase in the prevalence or frequency of cannabis use during the pandemic [26, 36, 55•, 57], five studies reported a decrease in cannabis use [28, 32•, 35, 39, 47], and three studies reported no change [24, 31, 34••]. Eight studies did not report an increase or decrease in cannabis use for similar reasons as mentioned above [25, 27, 33, 42, 44, 46, 54, 56]. Three studies included a mention of gender and two in relation to cannabis use [25, 28, 33]. One concluded that cannabis use among boys had increased more than use among girls during the pandemic [33], and one study reported that use among girls had increased more than for boys [28]. One study included an assessment of gender without relevance to cannabis use outcome [25].

### Tobacco Use

A total of 27 studies included measures on tobacco use, with all but two including measures on one or more other types of substance use [22, 23••, 25–27, 29, 30, 33, 34••, 35–48, 54, 58]. One study exclusively assessed nicotine dependence [59], and one study solely employed a general measure of smoking [9]. Seventeen studies employed a cross-sectional design [22, 25, 26, 33, 37–41, 44–46, 48, 54, 58–60] and 10 studies used longitudinal designs [23••, 27, 29, 30, 34••, 35, 36, 42, 43, 47]. Twenty studies used a non-random selection of participants, again including convenience, purposive, or volunteer samples [22, 25, 26, 29, 35–46, 54, 58–60].

Of all studies included for tobacco use, only two studies reported an increase in smoking behavior during the pandemic [26, 34••], six studies reported a decrease in smoking behavior [22, 23••, 35, 36, 39, 61], and one study reported no change in smoking behavior [47]. Eighteen studies did not report an increase or decrease in smoking behavior, again, mainly because of cross-sectional design and where smoking was employed as a covariate or group divider, or both [25, 27, 29, 30, 33, 37, 38, 40–46, 48, 54, 58, 59]; most of these studies focused on mental health. Nine studies reported on some form of gender difference [23••, 24, 33, 40, 41, 43, 47, 48, 59] but only two of them reported such difference in smoking, with one reporting increased use among boys [33] and one increased use for girls [48].

### E-cigarette Use/Vaping

A total of 16 studies included measures on e-cigarettes or vaping. Twelve of those also included measures into one or more other type of substance use [23••, 24–29, 32•, 34••, 36, 54, 58] but four were exclusively about e-cigarette use/vaping [62–65]. Nine of the studies employed a cross-sectional design [25, 26, 58, 59, 62–65] and eight used longitudinal designs [23••, 24, 27–29, 32•, 34••, 36]. Thirteen of the studies used a non-random selection of participants such as convenience, purposive, or volunteer samples [24–26, 28, 29, 36, 54, 58, 62–65].

One study reported an increase in e-cigarette use/vaping [26], eights studies reported a decrease in e-cigarette use/vaping [23••, 28, 36, 62–65], and two studies reported no change [24, 34••]. Six studies reported neither an increase nor a decrease in e-cigarette use/vaping [25, 27, 29, 32•, 54, 58]. Three studies included a mention of gender [23••, 25, 28] but only one in relation to e-cigarette use/vaping which reported non-significant gender differences in such use [23••].

### Use of Other Drugs and Unspecified Substance Use

A total of 19 studies included measures on other drugs or substance use without specification. Twelve of these studies employed a general measure of substance use or drug use [22, 25, 30, 40, 43, 45, 46, 66–70] without specification of substance but the remaining seven studies included measures on substances such as opioids/prescription drugs, heroin, cocaine, methamphetamine, and inhalants [27, 31, 33, 34••, 39, 42, 44]. Twelve studies employed a cross-sectional design [22, 25, 33, 39, 40, 44–46, 67–70] and seven used longitudinal designs [27, 30, 31, 34••, 42, 43, 67]. Fifteen studies used a non-random selection of participants such as via convenience, purposive, or volunteer samples [22, 25, 31, 39, 40, 42–46, 66–70].

Three studies reported increase in substance use [22, 27, 34••], three studies reported a decrease in use [39, 67, 67], and one study reported no change during the pandemic [31]. Twelve studies did not report an increase or decrease in substance use where such measures were primarily employed as covariates or group dividers [25, 30, 33, 40, 42–46, 68–70]. Four studies included a mention of gender [25, 33, 43, 70] but none of them in relation to differences in substance use.

## Discussion

The COVID-19 pandemic and associated social restrictions implemented to contain the spread of the virus have led to concerns from parents, educators, and healthcare professionals and researchers about what effects the pandemic may have had on the mental health and social well-being of youth. To partially address this concern, the objective of this systematic review was to examine the prevalence of youth substance use during the COVID-19 pandemic. Based on 49 studies published to date and captured in our search, the overall results of our review suggest that the prevalence of youth alcohol, cannabis, tobacco, and e-cigarette/vaping use has declined during the pandemic.

This finding of an overall decline in the prevalence of substance use during the pandemic is certainly positive, but it begs the question: *To what can the decrease be attributed?* Youth substance use most often takes place outside the home environment and usually within the context of the peer group. Moreover, youth substance use is highly dependent on availability and access to drugs and other substances. The public health restrictions that were necessary during the COVID-19 pandemic limited the time most adolescents spent in-person with their peers, and it follows that availability and access to alcohol, tobacco, and other substances was effectively limited during community lockdowns. In short, young people confined to their homes with parents had fewer opportunities for accessing and using substances. Thus, limited peer-group gatherings, decreased availability and access to substances, and increased time spent in the home with parents—all well-established factors shown to be effective in prevention efforts aimed at decreasing substance use [71]—are likely to have conferred important protection against substance use during COVID-19 as observed in the decline in prevalence reported across the bulk of studies we reviewed.

These promising and positive findings of an overall decrease in substance use, however, need to be viewed with some caution. First, some groups of youth may have had more pre-pandemic vulnerability to substance use during the pandemic for several reasons. For instance, there is evidence that mental health problems have been on the rise among many adolescents prior to and during the pandemic. In addition, for older adolescents and young adults experiencing increased stress and mental health problems, there is evidence that alcohol, drugs, and other substances may have offered a coping mechanism during the pandemic [12]. Youth that used substances by themselves, moreover, had increased symptoms of depression [28].

Spending more time in the household is not always a consistent protective factor. One study found that youth were drinking and using other substances with their parents shortly after social distancing measures were imposed, suggesting that permissive parental attitudes and behaviors could encourage and facilitate youth alcohol consumption and other substance use [72]. These permissive attitudes and modelling of health compromising behavior can influence the perceived norms towards substance use, resulting in increased use after the pandemic. Moreover, adolescents living with family conflict or dysfunction are more likely to engage in substance use [73]. One systematic review of 32 reports [74] found evidence that domestic violence has increased during the pandemic, indicating that the at-risk group of youth living with family conflict and dysfunction increased during this time. Finally, in addition, youth living under the stress of parental substance use, family dysfunction, and domestic violence could predispose the later onset of substance use and violent behavior. Youth who missed out on “normal teenage years” or important rites of passage that were interrupted by the pandemic may also have difficulties with substance use later in life when restrictions are removed, and social gatherings allowed. What this means for the prevalence of substance use in the post-pandemic years will require monitoring and further surveillance. Thus, the long-term effects of the pandemic and its potential dormant or latent effects on responsible adult substance use are unknown at this time and not likely to be fully understood until years later.

### Implications for Prevention and Treatment

Although the findings of our review suggest that the various mitigation strategies to contain the spread of COVID-19—masking, physical distancing, and community lockdowns that imposed restrictions on social gatherings—may have had detrimental impact on the mental health and social well-being of youth [21], such measures did not necessarily lead or contribute to an increase in youth substance use. Notwithstanding, there are several implications for prevention and treatment that should be considered in the aftermath of this pandemic. First, focusing on improving adolescent mental health should be a priority. Poor mental health is a well-known risk factor for substance use and misuse and the majority of young people with substance use problems suffer from co-occurring mental health issues that are often difficult to treat [75, 76]. Second, although remote learning enabled young people to maintain some connection to schooling, studies have pointed to the negative impact of virtual learning on the academic and social development of many young people and thus may have set the stage for a “lost generation” of youth who could be at even greater risk for substance use in the future [19]. Post-pandemic efforts undoubtedly will need to address the gaps in academic and social development of this cohort of young people—especially those for whom there have been significant disparities in access to educational opportunities. This suggests that community-wide surveillance and prevention of substance use needs to become a greater community priority than prior to the pandemic. Third, COVID-19 has demonstrated both the value of e-health and telemedicine to address the health needs of people during the pandemic [77]. However, the limited availability and access to mental health counseling and other forms of virtual treatment during the early phases of the COVID-19 pandemic may have contributed to placing young people at greater risk for substance use. As such, greater investment in e-health treatment for mental health problems and referral should be a greater priority in the future.

### Limitations

The findings of this review should be viewed with some caution because of design and other methodological limitations of the studies we reviewed. First, most of the published studies we reviewed utilized cross-sectional designs and focused largely on prevalence of use; few studies utilized longitudinal designs, outcome measures varied, and any follow-up was of limited time duration. Second, many studies used non-probability sampling methods to identify and obtain participants, including convenience, purposive, or volunteer samples, all of which limit the external validity of their findings. Third, few studies reported analyses that examined differences by gender. This remains an important question for future research because of the gender differences that have been observed in substance use and mental health outcomes during the COVID-19 pandemic [23••]. Finally, most of the studies reviewed included investigations of substance use of a single category, rather than across multiple categories of substance use, thus precluding analysis of any synergistic or gateway effects of multiple drug use for which the pandemic may have been responsible.

### Recommendations for Future Research

Our review suggests several directions for future investigation. First, numerous studies have now documented the impact of COVID-19 on the lives and well-being of adolescents in the immediate aftermath of the pandemic; however, more longitudinal studies are needed to assess the latent and long-term effect of the pandemic on substance use behaviors among youth. Although the pandemic may not have fostered increased substance use among most young people, further investigation is needed to understand differential risk across high-risk adolescents and differences by gender during the pandemic. In addition, more attention should be given to the role of key covariates in understanding youth substance use. For example, covariates such as socioeconomic status and social determinants of mental health should be addressed in research that seeks to understand the relationship of substance use to youth mental health and social well-being. Finally, as more studies are published, meta-analyses of youth substance use during and following the pandemic will be possible and are needed to better understand how and to what extent the pandemic influenced substance use and any underlying causal mechanisms.

## Conclusions

This systematic review of youth substance use during the COVID-19 pandemic assessed studies across several categories of substances, including alcohol, cannabis, tobacco, e-cigarette/vaping, and use of other drugs and unspecified substances. Regardless of the type of substance use, we found little evidence across the 49 studies we reviewed that the prevalence of use increased in response to the potential social and emotional deprivations associated with the pandemic. In fact, apart from some increase in the use of unspecified drugs or other substances, the majority of studies reported reductions in use across alcohol, cannabis, and tobacco and related products. Thus, we conclude that the bulk of the available evidence suggests that the prevalence of youth substance use largely declined during the first 2 years of the pandemic.

## Supplementary Information

Below is the link to the electronic supplementary material.

Supplementary file1 (XLSX 26 KB)
